# Detection of SARS-CoV-2 virus on the ocular surface of an asymptomatic health-care professional

**DOI:** 10.5935/0004-2749.20230047

**Published:** 2023

**Authors:** Luísa Grave Gross, Matheus Schwengber Gasparini, Leandro Mechi dos Santos, Ahmad Mohamad Ali Hamade, Arthur Pinheiro Favarato, Pierina Lorencini Parise, José Luiz Proença-Modena, Mônica Alves

**Affiliations:** 1 Department of Ophthalmology and Otorhinolaryngology, Faculdade de Ciências Médicas, Universidade Estadual de Campinas, Campinas, SP, Brazil; 2 Laboratory of Emerging Viruses, Department of Genetics, Microbiology and Immunology, Institute of Biology, Universidade Estadual de Campinas, Campinas, SP, Brazil; 3 Laboratory of Electrophysiology Neurobiology and Behavior, Universidade Estadual de Campinas, Campinas, SP, Brazil

**Keywords:** Coronavirus infection, COVID-19, Eye Infection, viral, SARS-CoV-2, Health professional, Infecção por coronavírus, COVID-19, Infecção ocular viral, SARS-CoV-2, Profissional de saúde

## Abstract

Coronavirus disease (COVID-19) is an infectious disease caused by severe acute respiratory syndrome coronavirus 2 (SARS-CoV-2). Its main form of transmission is through respiratory droplets. Case reports have described the presence of this virus in biological materials such as blood, feces, urine, and tears, which generate hypotheses about other means thereby the disease is transmitted. In this report, we describe a case of SARS-CoV-2 identified on the eye surface of an asymptomatic health-care professional. The nasopharyngeal reverse transcription polymerase chain reaction test, using a sample collected on the same day, and the serological test, performed 3 months later, did not reveal any evidence of SARS-CoV-2 infection. These results alert on the possibility of a false-positive reverse transcription polymerase chain reaction result for the ocular surface or the presence of the virus in the conjunctival mucosa in individuals without infection.

## Abstract (pt)

A COVID-19 é uma doença infeciosa causada pelo SARS-CoV-2, sendo sua principal forma de transmissão através de gotículas respiratórias. Já existem relatos de caso descrevendo a presença desse vírus em materiais biológicos como sangue, fezes, urina e lágrima, o que gera hipóteses sobre outros meios de transmissão da doença. Neste estudo, descrevemos um caso de identificação do vírus SARS-CoV-2 na superfície ocular de um profissional de saúde assintomático. A transcrição inversa da reação em cadeia da polimerase da nasofaringe, coletada no mesmo dia, e o teste sorológico, realizado três meses após, não detectaram qualquer evidência de infecção pelo SARS-CoV-2. Esses dados alertam para a possibilidade de resultado falso positivo da transcrição inversa da reação em cadeia da polimerase da superfície ocular ou a presença do vírus na mucosa conjuntival sem infecção.

## INTRODUCTION

Coronavirus disease (COVID-19) is an infectious disease caused by severe acute respiratory syndrome coronavirus 2 (SARS-CoV-2)^(1)^. Although the main form of transmission is through respiratory droplets, case reports have described the presence of SARS-CoV-2 in biological materials such as blood, feces, urine, and tears, which generates hypotheses about other means by which the disease is transmitted^(2)^. Alternative sites for RT-PCR sample collection have been studied, including the conjunctival mucosa, as it is an easily accessible site^(3,4)^.

In this report, we describe a case of SARS-CoV-2 identified on the eye surface of an asymptomatic health-care professional. The nasopharyngeal RT-PCR test, using a sample collected on the same day, and the serological test, performed 3 months later, did not reveal any evidence of SARS-CoV-2 infection. These data alert on the possibility of a false-positive RT-PCR test result for the ocular surface or the presence of the virus in the conjunctival mucosa in individuals without the infection.

## CASE REPORT

During the COVID-19 pandemic, five ophthalmology residents participated in a clinical study involving the collection of ocular secretion from 83 patients with COVID-19 who were hospitalized in a ward or the intensive care unit at Hospital de Clínicas, *Universidade Estadual de Campinas* (UNICAMP). Samples were collected from July 7, 2020, to August 11, 2020, using the appropriate personal protective equipment, consisting of a N95 mask, a face shield, sterile gloves, a surgical cap, and a disposable cloak, in accordance with the COVID-19 prevention protocols for health-care professionals.

On August 4, 2020, 28 days after the beginning of the collection of the ocular secretion samples from the hospitalized patients, nasopharyngeal and ocular surface samples were collected from the physicians for RT-PCR analysis. The physicians were asymptomatic and had shown no clinical evidence of COVID-19 since the start of the pandemic. They were aged between 20 and 30 years and did not have any systemic or ophthalmic comorbidity.

Ocular surface material was collected by smearing the lower fornix of the conjunctiva in both eyes with a swab, without topical anesthesia. Nasopharyngeal material was obtained by smearing the region with a swab. The collected samples were stored with PrimeStore Molecular Transport Medium at room temperature for up to 7 days and at -80°C after this period until RNA extraction with the Extracta kit for RNA and Loccus for viral DNA using the automated extraction equipment Thermo Scientific KingFisher Flex Purification System. RT quantitative PCR (qPCR) analysis was performed for the detection of the viral genome with the GeneFinder kit COVID-19 Plus RealAmp Kit, in accordance with the manufacturer’s instructions.

The laboratory results demonstrated the absence of SARS-CoV-2 in the nasopharynx samples of the five subjects. However, the virus was detected in the eye sample of one of the subjects (Table 1), who was positive for viral gene E (Ct=18.99), gene N (Ct=16.78), and gene RpRd (Ct=19.41). The subject remained asymptomatic in the following weeks. Afterward, a high viral load (1.33 x 10^4^ plaque-forming unit equivalents/mL) for SARS-CoV-2 was detected in the tear sample of the subject after comparison with a standard curve using the RT-qPCR Charité protocol (Table 1). However, isolation of SARS-CoV-2 was not possible because the sample transport medium only preserves the genetic material but inactivates the virus.

**Table 1:** Cycle threshold (Ct) values obtained from the ocular surface RT-PCR assay results of the health-care professionals

Subject	Gene finder protocol			Charité protocol
	Gene E	Gene N	Gene RpRd	Quantity (PFU eq/mL)
*A	**18.99**	**16.78**	**19.41**	**1.33 x 10^4**
**B**	>45	>45	>45	
**C**	>45	>45	>45	
**D**	>45	>45	>45	
**E**	>45	>45	>45	

*Ophthalmology resident with SARS-CoV-2 detected on the ocular surface.

Gene E= envelope; Gene N= nucleoprotein; Gene RdRp= RNA-dependent RNA polymerase; RT-PCR= real-time polymerase chain reaction; PFU eq/mL= plaque-forming units from viral RNA equivalents per milliliter.

On November 19, 2020, 3 months and 15 days after the swab samples were collected, all the subjects underwent a serological test for detection of antibodies against SARS-CoV-2 (SARS-CoV-2 IgG Kit, Abbott Chemiluminescent Microparticle Immunoassay), with negative results (Table 2).

**Table 2:** Results of the detection of antibodies against SARS-CoV-2 in the health-care professionals

Subject	CMIA IgG PURE	CMIA IgG PURE (S/CO)	CMIA IgM PURE	CMIA IgM PURE (S/CO)
*A	Negative	0.08	Negative	0.12
**B**	Negative	0.03	Negative	0.12
**C**	Negative	0.03	Negative	0.05
**D**	Negative	0.13	Negative	0.1
**E**	Negative	0.03	Negative	0.23

*Ophthalmology resident with SARS-CoV-2 detected on the ocular surface.

CMIA= chemiluminescent microparticle immunoassay

CMIA IgG or IgM: <0.9, negative; 0.9-1.4, inconclusive; and >1.4, positive.

## DISCUSSION

The COVID-19 diagnosis is based on the detection of viral RNA through a RT-PCR analysis using a nasopharyngeal swab sample. Alternative sites of easy access and low discomfort have been studied for sample collection, such as the conjunctival mucosa^(3,4)^. Recently, both direct infection of the ocular surface and viral transmission through the nasolacrimal duct to the nasal epithelium have been suggested to be plausible^(5)^. Thus, the conjunctival mucosa stands out as an alternative collection site to the nasopharyngeal mucosa.

SARS-CoV-2 can be detected 1 to 2 days before the onset of respiratory symptoms and can persist from 7 to 12 days in moderate cases and up to 2 weeks in severe cases^(6)^. While RT-PCR can be used to detect viral nucleic acid, serological tests are useful for the detection of antibodies in blood circulation in subjects with the infection. In IgM and IgG serological tests for COVID-19, antibodies can be detected from 5 and 14 days after the onset of symptoms, respectively^(7)^. These results are similar to the antibody dosages in other acute viral infections.

Nasopharyngeal RT-PCR is considered the gold standard test for SARS-CoV-2 detection. Data obtained in vitro, together with clinical data, suggest that this test has a high specificity (approximately 98.8%) but moderate sensitivity, ranging from 63% to 78%^(8)^. Although the sensitivity and specificity values of serological tests are high, ranging from 72.7% to 100% and from 98.7% to 100%, respectively, a large variability exists between the kits available in the market. To date, none of these kits have been approved for use alone to confirm or exclude COVID-19 infection^(9)^.

In this report, we describe a case of SARS-CoV-2 identified on the eye surface of an asymptomatic health-care professional. The RT-PCR test of the nasopharynx, using a sample collected on the same day, and the serological test performed later did not reveal any evidence of SARS-CoV-2 infection. The literature provides no data on the sensitivity and specificity of ocular surface detection using RT-PCR. However, considering the high specificity of the test when using nasopharyngeal samples, the possibility of a false-positive RT-PCR test result for the ocular surface is less likely than the hypothesis that the virus is present in the conjunctival mucosa in the absence of infection.

This case report raises important considerations about the presence of SARS-CoV-2 on the ocular surface. RT-PCR analysis of conjunctival samples may yield false-positive results, which impairs its accuracy. Further studies are needed to determine the percentage of falsepo sitive and false-negative results and, consequently, the real accuracy of RT-PCR as a diagnostic alternative for COVID-19. In addition, in individuals exposed to patients with SARS-CoV-2 infection, the virus may be present in the conjunctival mucosa without detection of the infection using conventional methods or based on clinical symptoms of COVID-19. This implies a possible route of virus contamination and raises important questions about biosafety.
